# Physiologic Characteristics of Hyperosmolar Therapy After Pediatric Traumatic Brain Injury

**DOI:** 10.3389/fneur.2021.662089

**Published:** 2021-04-20

**Authors:** Jeffrey Wellard, Michael Kuwabara, P. David Adelson, Brian Appavu

**Affiliations:** ^1^University of Arizona College of Medicine–Phoenix, Phoenix, AZ, United States; ^2^Department of Radiology, Phoenix Children's Hospital, Phoenix, AZ, United States; ^3^Department of Neurosciences, Barrow Neurological Institute at Phoenix Children's Hospital, Phoenix, AZ, United States

**Keywords:** hyperosmolar therapy, hypertonic saline, mannitol, traumatic brain injury, pediatrics, cerebrovascular pressure reactivity, multimodality monitoring

## Abstract

All work was performed at the Barrow Neurological Institute at Phoenix Children's Hospital.

**Objective:** Investigate injury severity, neuroimaging, physiology, and outcomes with bolus hyperosmolar therapy (HT) of 3% hypertonic saline or mannitol.

**Methods:** Retrospective cohort analysis was performed. Physiologic variables included intracranial pressure (ICP), arterial blood pressure (ABP), and heart rate (HR). Volume-pressure compensation (PVC) indices included ICP pulse amplitude (AMP) and correlation of AMP and ICP (RAP). Cerebrovascular pressure reactivity (CVPR) indices included pressure reactivity index (PRx), pulse amplitude index (PAx), wavelet PRx (wPRx), and correlation of AMP and cerebral perfusion pressure (RAC). Heart rate variability (HRV) indices included heart rate standard deviation (HRsd), heart rate root mean square of successive differences (HRrmssd) and low-high frequency ratio (LHF). Outcome was assessed using Glasgow Outcomes Scale Extended Pediatrics, 12-months post-injury. Generalized estimating equations was applied to investigate associations of physiologic changes and pre-treatment indices with HT efficacy. Repeated measures analysis of variance was applied to investigate changes after HT without intracranial hypertension (ICH). Wilcoxon rank-sum was applied to investigate HT responsiveness with age, injury severity, neuroimaging, and outcomes.

**Results:** Thirty children received bolus HT. ICH reduction after HT was associated with reduced ICP (*p* = 0.0064), ABP (*p* = 0.0126), PRx (*p* = 0.0063), increased HRsd (*p* = 0.0408), and decreased pretreatment RAC (*p* = 0.0115) and wPRx (*p* = 0.0072). HT-responsive patients were older and had improved outcomes (*p* = 0.0394). HT without ICH was associated with increased ICP (*P* < 0.0001) and ABP (*P* < 0.0001), increases in all HRV indices and decreases in all PVC indices.

**Conclusion:** After pediatric TBI, efficacious HT is associated with decreased ICP and ABP, pre-treatment indices suggesting efficient CVPR, and potentially improved outcomes.

## Introduction

Traumatic brain injury (TBI) represents a leading cause of pediatric morbidity and mortality, affecting an estimated 280 per 100,000 children in the United States (1). Despite the significance of this condition, high-level recommendations are not available in current pediatric guidelines, with no level I evidence existing to guide clinical management (2). While most level II evidence focuses on therapies to avoid in care, level II evidence does exist to support bolus therapy of hypertonic saline (3%) for treatment of intracranial hypertension (ICH). Mannitol is also available as a hyperosmolar agent, but it has not been subjected to rigorous controlled clinical trials vs. placebo or other therapy in children. After TBI, ICH can arise from multiple sources including cerebral edema, increased cerebral arterial blood volume (CABV), increased cerebral sinus pressure, or obstructive hydrocephalus. While use of hyperosmolar therapy (HT) has been shown to reduce ICH, its putative pharmacological mechanisms focus on reduction of cerebral edema, yet this would make it potentially ineffective when ICH arises from other etiologies. Furthermore, concern has arisen that in patients with intact cerebrovascular pressure reactivity (CVPR), hypertonic saline may impair cerebral hemodynamics (3). For these reasons, further study and understanding of the mechanisms of actions of HT and real time responses to treatment are necessary.

Multimodality neurologic monitoring (MMM) represents an emerging technique to help clinicians understand complex physiologic states after TBI (4). This technique offers both data visualization of real-time physiology, as well as calculation of biomarkers of cerebral dynamics. Model-based indices of CVPR, such as the pressure reactivity index (PRx), have been developed to understand a patient's state of pressure-related cerebral autoregulation, and have been demonstrated to be a strong marker of injury severity after pediatric TBI (5–7). Other model-based indices of heart rate variability (HRV) and pressure-volume compensation (PVC) offer an opportunity to better characterize a patient's physiologic state (8–10). These calculated biomarkers, however, have not yet been recognized to offer effective and immediately actionable information to guide clinical care.

In this study, we wished to investigate physiologic changes associated with HT in pediatric TBI patients, as well as the association of HT efficacy for ICH with physiologic, patient-specific, and neuroimaging characteristics. We hypothesized that HT is associated with reduction of ICH. We also hypothesized that HT efficacy for ICH is associated with pre-treatment CVPR and that treatment efficacy is associated with changes in CVPR, PVC, HRV as well as improved functional outcomes.

## Materials and Methods

This was a retrospective study from a prospectively collected clinical database. The study was conducted at Phoenix Children's Hospital (Phoenix Arizona, USA) and was approved by the Institutional Review Board (17-469).

Children (<21 years of age) with TBI from a single pediatric intensive care unit who underwent continuous intraparenchymal intracranial pressure (ICP) monitoring and MMM were retrospectively analyzed from September 2014 to December 2019. Patients were managed according to institutional standard of care, which consisted of invasive arterial blood pressure (ABP) and invasive intracranial pressure monitoring with an intraparenchymal probe and sometimes an external ventricular drain (EVD). The decision to place an intraparenchymal probe and/or EVD was made based on the risk/benefit ratio by the neurosurgeon on call. Clinical care was implemented using an institutional protocol founded upon the most current pediatric TBI guidelines at the time (2, 11).

The primary aim of this study was to investigate physiologic changes associated with bolus HT. We classified patients who had evidence of ICH during the immediate hour prior to bolus HT, as well as patients who had no evidence of ICH during that period. Evidence of ICH was classified using a threshold of a dose of ICP > 20 mmHg within the hour preceding treatment that was >0 mmHg/h. We calculated dose of ICH using a previously described methodology accounting for cumulative extent and duration identified by the area under the curve above the threshold, ICP >20 mmHg over a 1 h period, obtaining measurements of mmHg/hour (12). In patients without pre-treatment evidence of ICH, we compared physiologic changes in the hour immediately after HT as compared to the hour immediately preceding treatment. In patients with evidence of ICH, we compared physiologic changes associated with treatment efficacy as compared to changes associated with treatment inefficacy. We also investigated the association of treatment efficacy with pre-treatment serum sodium levels and model-based indices of CVPR, PVC and HRV. Secondary aims of this study were to investigate whether treatment efficacy of HT was associated with patient characteristics such as injury severity, initial neuroimaging characteristics and global functional outcomes.

### Patient Characteristics

Demographic patient information included age. Injury characteristics included Glasgow Coma Scale (GCS) score (13) at presentation in addition to the Pediatric Risk of Mortality III (PRISM III) score (14) on day of admission. GCS scores range from 1 to 8 with lower scores indicative of increased injury severity. PRISM III scores range from 0 to 74 with higher scores indicative of increased injury severity. Global functional outcome was measured using the Glasgow Outcome Score Extended-Pediatrics (GOSE-PEDs) score at 12 months after injury (15), GOSE-PEDs scores range from 1 to 8 with higher values representing worsened outcome.

### Physiologic Data

Patients underwent MMM that included intraparenchymal ICP, invasive ABP (arterial blood pressure) and electrocardiogram (ECG) monitoring. Cerebral perfusion pressure (CPP) was calculated as the intraparenchymal ICP subtracted from the mean ICP. All patients also underwent continuous electroencephalography (cEEG) as part of MMM. Continuous physiologic data from all monitoring devices were collected and time-synchronized using an MMM device (CNS200; Moberg ICU Solutions, Philadelphia, PA). ICM+ software (Cambridge, UK) was used to visualize and process MMM data to calculate model-based indices of CVPR, PVC, and HRV.

Cerebral hemodynamic variables collected for this study included intracranial pressure (ICP), arterial blood pressure (ABP) and heart rate (HR). Median dose of each hemodynamic variable was computed in the hour before and after bolus HT, and the difference after treatment was computed for each variable. Model-based indices utilized in this study were computed based upon each of these hemodynamic variables.

Model based indices of CVPR, PVC, and HRV are described in Supplementary Table 1. We classify model based indices as calculated values of two or more physiologic data captured from time series MMM signals that have been developed from conceptual models that describe physiologic states. Four different CVPR indices were explored, including the pressure reactivity index (PRx), pulse amplitude index (PAx), wavelet PRx (wPRx), and correlation of cerebral perfusion pressure and ICP pulse amplitude (RAC). A higher value for these indices signified impaired CVPR. PRx was calculated as a moving Pearson correlation coefficient between ABP and ICP within a 5-min averaging window (16). PAx was calculated similarly as a moving correlation coefficient between ABP and ICP pulse amplitude (17). RAC was calculated similarly as a moving correlation coefficient of ICP pulse amplitude and CPP (18). WPRx was calculated by taking the cosine of the wavelet transform phase shift between ABP and ICP (19). Physiologic indices of PVC included ICP pulse amplitude (AMP) and the correlation coefficient of AMP and ICP index (RAP). AMP represents the amplitude of the ICP waveform. RAP is calculated as a moving Pearson correlation coefficient between ICP within a 5-min averaging window (10). Indices of HRV included standard deviation of heart rate (HRsd), root-mean square of successive differences in heart rate (HRrmssd), and heart rate low-high frequency ratio (LHF). A lower value for these indices signifies impaired autonomic function. HRV variables were computed from time- and frequency-domain analyses according to international guidelines (8). For all HRV variables, a 30 s time series of R-R intervals was assessed from ECG that was updated every 10 s. HRsd and HRrmssd were computed from the time-domain signal. LHF was computed in the frequency domain, using Lomb-Scargle periodogram to calculate the spectral power of the RR time series in the low frequency range (0.04–0.15 Hz) and the high frequency range (0.15–0.4 Hz). To explore other physiologic biomarkers that may explain efficacy of HT based on its mechanism, we evaluated serum sodium levels acquired prior to HT.

Physiologic biomarkers investigated in relation to treatment efficacy were collected during the 1 h epoch immediately preceding and then proceeding bolus HT. The dose of ICH was also calculated in the 1 h immediately before and after bolus HT as the area under the curve during that time interval when ICP was >20 mmHg and was recorded in units of mmHg/hour, as done in previously described methods (12). Treatment efficacy was defined as a reduction in dose of ICP > 20 mmHg from the 1 h after initiation of bolus therapy as compared to the hour prior to bolus therapy. For example, a reduction from pre-treatment dose of ICH of 2.5 mmHg/h to a post-treatment dose of 1.8 mmHg/h would represent treatment efficacy. In contrast, a rise from a pre-treatment dose of ICH of 350.6 mmHg/h to a post-treatment dose of 355.6 mmHg/h would represent treatment inefficacy. The assessment of treatment efficacy was thus only evaluated for boluses which were preceded by ICP achieving a threshold dose of ICP >20 mmHg >0 mmHg/h. ICH Agents used for bolus dosing of HT included 3% hypertonic saline and mannitol. To characterize treatment responsiveness at the subject level, individual patients were considered responsive to HT if they experienced treatment efficacy to ≥50% of the bolus HT given to them during their monitoring.

### Neuroimaging Characteristics

Initial computed tomography (CT) scans were reviewed by a neuroradiologist in their original digital form and graded according to the modified Marshall CT scan classification system (20). The neuroradiologist was blinded to MMM data or outcome measures at the time of analysis. Volumes of high- or mixed-density lesions consistent with intracranial hematomas were calculated by creating volumetric three-dimensional rendering using a commercially available region growing segmentation algorithm (Royal Phillips®, Best, Netherlands). Midline shift was measured in millimeters at the level of maximum deviation. The presence of basal cistern effacement was also described for each patient.

### Statistical Analysis

Continuous and categorical variables were summarized with descriptive statistics including mean and standard deviation (SD) or median and interquartile range (IQR) of continuous variables, and frequencies of categorical variables. In patients with pre-treatment ICH, we employed generalized estimating equations (GEE), accounting for repeated measures (21), to test for the association of HT efficacy with differences in continuous physiologic variables. We also employed GEE in the same cohort to investigate the association of treatment efficacy with pre-treatment sodium levels and model-based indices of CVPR, PVC, and HRV. In patients without pre-treatment ICH, we employed one-way repeated measures analysis of variance (ANOVA) to test for changes in continuous physiologic variables before and after HT (22). Wilcoxon-rank sum test was used to investigate the association of continuous patient and neuroimaging characteristics with treatment responsiveness after HT. Fisher's exact test was used to test for the association of the presence or absence of basal cistern effacement or inflicted head trauma with treatment responsiveness. Findings were considered significant if the *p* < 0.05. Statistical analyses were performed using RStudio version 3.4.1.

## Results

### Population

Seventy-nine children were identified with TBI undergoing MMM with ICP monitoring, among which seventy-four children (93.7%) underwent intraparenchymal ICP monitoring. Of those seventy-four children, thirty (40.5%) were identified to have received distinct boluses of HT during their care with MMM. Table 1 summarizes patient demographic data regarding TBI patients who received bolus HT. Ten patients (33.3%) were female. Ages ranged from 1 month to 16 years of age (median 6.5+/– interquartile range [IQR] 11.5). Nine of the thirty patients (30.0%) who underwent bolus HT had inflicted head injuries, among which five (55.6%) experiencing abusive head trauma and four (44.4%) experiencing gunshot wounds to the head. Initial GCS scores ranged from 3 to 15 (median 6.5 ± 4.8). Initial PRISM III scores ranged from 9 to 25 (median 16.5 ± 7.0). GOSE-PEDs scores at 12 months ranged from 1 to 8 (median 5.0 ± 3.0). Three patients (10.0%) experienced medically and surgically refractory ICH and died after withdrawal of life sustaining therapies. Serum sodium levels prior to bolus HT ranged from 138 to 175 mmol/L (median 148.0 ± 6.0). Twenty-two of the thirty patients (73.3%) receiving bolus HT had initial CT scans available for review. Initial CT Marshall scores ranged from 1 to 6 (median 2.0 ± 4.0). Intracranial hematoma volume ranged from 0 to 143.7 cm^3^ (median 17.8 ± 25.8). Midline shift ranged from 0 to 21 mm (median 1.0 ± 2.8). Six patients (27.3%) demonstrated evidence of basal cistern effacement. All patients received intravenous Levetiracetam for seizure prophylaxis and nine patients (30.0%) experienced post-seizures, although seizures and antiepileptic treatment administration were not identified during the hour before or after HT for observed patients.

**Table 1:** Patient characteristics.

**Characteristic (*n* = 30)**	**Number (%) of patients**
Female, number (%)	10 (33.3)
Mortality	3 (10.0)
3% Hypertonic saline, exclusively	17 (56.7)
Mannitol, exclusively	5 (16.7)
3% hypertonic saline and mannitol	8 (26.7%)
Treatment Responders	7 (23.3%)
Presence of basal cistern effacement	6 (27.3%)
	**Median (IQR)**
Age	6.5 (11.5)
Initial Glasgow Coma Score	6.5 (4.8)
Initial PRISM III Score	16.5 (7.0)
GOSE-PEDs, 12 months	5.0 (3.0)
Na level prior to therapy, mmol/L	148.0 (6.0)
CT Marshall Score	2.0 (4.0)
Intracranial Hematoma Volume (cm^3^)	17.8 (25.8)
Midline shift (mm)	1.0 (2.8)
Bolus therapies per patient	3.0 (4.8)
3% Hypertonic saline dose, mL/kg	5.8 (2.5)
Mannitol dose, gm/kg	0.3 (0.1)
Timing of bolus hyperosmolar therapy after initial injury (days)	2.0 (3.0)

*Note that values “prior to hyperosmolar therapy” reflect the 1 h prior to therapy. Abbreviations: N, count; Na, sodium; GOSE-PEDs, Glasgow Outcome Scale Extended Pediatrics; PRISM, Pediatric Risk of Mortality; CT, computed tomography, mL, milliliters; gm, grams; kg, kilograms; %, percent; IQR, interquartile range*.

### Characteristics of Bolus Hyperosmolar Therapy and Intracranial Hypertension

Characteristics are also summarized in Table 1. There were 148 boluses of HT identified during MMM, with each patient receiving between 1 and 21 distinct boluses of therapy (median 3.0 ± 4.8). Boluses of HT were obtained from 0 to 13 days after initial injury (median 2.0 ± 3.0). Seventeen patients (56.7%) received boluses exclusively with 3% hypertonic saline, five patients (16.7%) received boluses exclusively with mannitol, and eight patients (26.7%) received both mannitol and 3% hypertonic saline. Bolus dose of 3% hypertonic saline ranged from 0.6 to 13.3 mL/kg (median 5.8 ± 2.5). Bolus dose of mannitol ranged from 0.2 to 0.7 g/kg (median 0.3 ± 0.1). Of the 148 distinct boluses of HT, 78 boluses were preceded by evidence of ICH (52.7%) in the 1 h prior to treatment. Of these 78 treatments, 44 boluses (56.4%) were followed by reduction of ICH, representative of treatment efficacy. Seven patients (23.3%) had ≥50% treatment responsiveness and thus were considered responsive to HT.

### Patient and Neuroimaging Characteristics Associated With Hyperosmolar Treatment Efficacy

Findings are summarized in Table 2. In patients with pre-treatment ICH, we observed that older age was associated with treatment efficacy (median 14.0±6.0 years; *p* = 0.0194) as compared to treatment inefficacy (median 4.0 ± 10.5 years). We did not observe associations of treatment efficacy with initial GCS or PRISM III scores, inflicted head trauma, CT Marshall scores, hematoma volume, midline shift, or the presence of basal cistern effacement. We did observe that patients who experienced treatment responsiveness had improved functional outcomes (median GOSE-PEDs 2.0 ± 3.5; *p* = 0.0394) at 12-months post-injury as compared to patients who did not experience treatment responsiveness (median GOSE-PEDs 5.0 ± 2.5).

**Table 2:** Pre-treatment characteristics associated with reduction of intracranial hypertension after hyperosmolar therapy.

**Biomarker**	**Effective [median (IQR)]**	**Ineffective [median (IQR)]**	***p*-value**
**Age, years**	**14.00 (6.00)**	**4.00 (10.50)**	**0.0194**
GCS score at presentation	4.00 (4.00)	7.00 (4.00)	0.2345
PRISM III score at presentation	15.00 (5.00)	17.00 (7.00)	0.9407
**GOSE-PEDs, 12 months**	**2.00 (3.50)**	**5.00 (2.50)**	**0.0394**
CT Marshall score	2.00 (2.00)	3.00 (4.00)	0.5907
Hematoma volume (cm^3^)	19.50 (21.90)	16.00 (15.40)	0.8907
Midline shift (mm)	2.00 (2.50)	0.00 (3.00)	0.8794
Serum Na (mmol/L)	148.00 (6.00)	148.00 (6.00)	0.0885
PRx, median	0.04 (0.43)	0.21 (0.86)	0.1148
PAx, median	−0.30 (0.37)	−0.13 (0.52)	0.1071
**wPRx, median**	**−0.09 (0.28)**	**0.04 (0.71)**	**0.0064**
**RAC, median**	**−0.61 (0.38)**	**−0.55 (0.61)**	**0.0115**
RAP, median	0.81 (0.29)	0.76 (0.43)	0.1764
AMP, median	1.62 (1.65)	1.87 (1.72)	0.3239
HRsd, median	2.62 (2.60)	1.86 (2.98)	0.4606
HRrmssd, median	15.00 (16.11)	5.78 (18.21)	0.2517
LHF Ratio, median	2.11 (2.08)	1.56 (2.30)	0.9469
	**Number (%) of patients**	**Number (%) of patients**	
Presence of basal cistern effacement	6 (27.3%)	16 (72.7%)	0.1206
Inflicted head trauma	4 (25.0%)	5 (35.7%)	0.3932

*Analysis of age, GCS, PRISM III, CT Marshall Score, hematoma volume, and midline shift performed using Wilcoxon rank sum test. Analysis of basal cistern effacement performed using Fisher's exact test. Analysis of all other variables performed using generalized estimating equations. Bold variables are representative of statistical significance. IQR, interquartile range; GCS, Glasgow Coma Scale; PRISM, Pediatric Risk of Mortality; GOSE-PEDs, Glasgow Outcome Scale –Extended Pediatrics; CT, computed tomography; cm, centimeters, mm, millimeters; Na, sodium; mmol/L, millimoles per liter; mmHg, millimeters of mercury; PRx, pressure reactivity index; wPRx, wavelet-pressure reactivity; PAx, pulse amplitude index; RAC, correlation coefficient between intracranial pressure pulse amplitude and cerebral perfusion pressure; RAP, correlation coefficient between intracranial pressure pulse amplitude and intracranial pressure; AMP, intracranial pressure pulse amplitude; HRsd, standard deviation of heart rate; HRrmssd, root mean square of successive differences of heart rate; LHF, low high frequency ratio*.

### Pre-treatment Sodium Levels and Model-Based Indices Associated With Hyperosmolar Treatment Efficacy

Findings are also summarized in Table 2. We observed that HT efficacy was associated with decreased pre-treatment median wPRx (0.05 *p* = 0.0072) and RAC (*p* = 0.0115) values. We did not observe that treatment efficacy was associated with pre-treatment serum sodium levels or values of PRx, PAx, RAP, AMP, HRsd, HRrmssd, or LHF Ratio.

### Physiologic Changes After Hyperosmolar Treatment With Preceding Intracranial Hypertension

Findings are summarized in Table 3. As expected, we observed that HT efficacy for ICH was associated with decreased median ICP, whereas treatment inefficacy was associated with increased median ICP (*p* = 0.0064). We observed that treatment efficacy was associated with decreased median ABP, whereas treatment unresponsiveness was associated with increased ABP (*p* = 0.0136). In terms of indices of CVPR, we observed that treatment efficacy was associated with decreases in median PRx, whereas treatment inefficacy was associated with increases in median PRx (*p* = 0.0063). We observed that treatment efficacy was associated with a greater decrease in median HRsd as compared to treatment inefficacy (*p* = 0.0408). We did not observe significant changes in relation to treatment responsiveness with regards to median HR, PAx, wPRx, RAC, RAP, AMP, HRrmssd, or LHF Ratio.

**Table 3:** Differences in cerebral physiology to bolus hyperosmolar therapy after intracranial hypertension.

**Physiologic variable**	**Difference with treatment responsiveness [median (IQR)]**	**Difference with treatment unresponsiveness [median (IQR)]**	***p*-value**
**ICP, median (mmHg)**	**−1.66 (3.38)**	**+2.14 (2.910)**	**0.0064**
**ABP, median (mmHg)**	**−0.67 (4.19)**	**+0.31 (5.26)**	**0.0126**
HR, median (bpm)	−1.00 (7.64)	+0.32 (6.38)	0.8903
**PRx, median**	**−0.04 (0.22)**	**+0.02 (0.24)**	**0.0063**
PAx, median	−0.06 (0.25)	+0.02 (0.25)	0.0725
wPRx, median	−0.03 (0.27)	−0.01 (0.25)	0.2610
RAC, median	−0.01 (0.17)	−0.03 (0.19)	0.3672
RAP, median	−0.01 (0.31)	+0.03 (0.11)	0.0756
AMP, median	−0.10 (0.40)	+0.16 (0.46)	0.0795
**HRsd, median**	**−0.06 (0.83)**	**−0.03 (0.40)**	**0.0408**
HRrmssd, median	−0.38 (5.06)	+0.04 (2.56)	0.5242
LHF Ratio, median	+0.05 (1.08)	+0.00 (0.29)	0.8476

*Analysis performed using generalized estimating equations. Bold variables are representative of statistical significance. ICP, intracranial pressure; ABP, arterial blood pressure; HR, heart rate; bpm, beats per minute; mmHg, millimeters of mercury; PRx, pressure reactivity index; PAx, pulse amplitude index; wPRx, wavelet-pressure reactivity; RAC, correlation coefficient between intracranial pressure pulse amplitude and cerebral perfusion pressure; RAP, correlation coefficient between intracranial pressure pulse amplitude and intracranial pressure; AMP, intracranial pressure pulse amplitude; HRsd, standard deviation of heart rate; HRrmssd, root mean square of successive differences of heart rate; LHF, low frequency-high frequency ratio*.

### Physiologic Changes After Hyperosmolar Treatment Without Preceding Intracranial Hypertension

Findings are summarized in Table 4. In terms of physiologic vital signs, we observed that HT is associated with increases in ICP (*p* < 0.0001) and ABP (*p* < 0.0001) and decreases in HR (*p* < 0.0001). In terms of indices of CVPR, we observed that HT is associated with increases in PRx (*p* < 0.0001) and decreases in PAx (*p* = 0.0005), wPRx (*p* < 0.0001), and RAC (*p* < 0.0001). In terms of indices of PVC, we observed that HT is associated with increases in RAP (p < 0.0001) and AMP (*p* < 0.0001). In terms of HRV, we observed that HT is associated with increases in HRsd (*p* < 0.0001), HRrmssd (*p* < 0.0001), and LHF ratio (*p* < 0.0001).

**Table 4:** Differences in cerebral physiology in relation to hyperosmolar therapy without intracranial hypertension.

**Physiologic variable**	**Prior to treatment [mean (SD)]**	**After treatment [mean (SD)]**	***p*-value**
**ICP, median (mmHg)**	**11.06 (4.96)**	**12.81 (10.49)**	**<0.0001**
**ABP, median (mmHg)**	**77.78 (11.25)**	**78.57 (11.07)**	**<0.0001**
**HR, median (bpm)**	**99.87 (29.35)**	**97.22 (31.19)**	**<0.0001**
**PRx, median**	**0.09 (0.41)**	**0.10 (0.40)**	**<0.0001**
**PAx, median**	**−0.22 (0.37)**	**−0.24 (0.36)**	**0.0005**
**wPRx, median**	**0.04 (0.35)**	**0.03 (0.36)**	**<0.0001**
**RAC, median**	**−0.35 (0.42)**	**−0.38 (0.40)**	**<0.0001**
**RAP, median**	**0.46 (0.39)**	**0.48 (0.38)**	**<0.0001**
**AMP, median**	**1.21 (1.17)**	**1.26 (1.15)**	**<0.0001**
**HRsd, median**	**3.11 (1.82)**	**3.38 (2.39)**	**<0.0001**
**HRrmssd, median**	**18.75 (18.43)**	**20.89 (19.46)**	**<0.0001**
**LHF Ratio, median**	**1.59 (1.56)**	**2.95 (10.81)**	**<0.0001**

*Analysis performed using one-way repeated-measures analysis of variance. Bold variables are representative of statistical significance. ICP, intracranial pressure; ABP, arterial blood pressure; HR, heart rate; bpm, beats per minute; mmHg, millimeters of mercury; PRx, pressure reactivity index; PAx, pulse amplitude index; wPRx, wavelet-pressure reactivity; RAC, correlation coefficient between intracranial pressure pulse amplitude and cerebral perfusion pressure; RAP, correlation coefficient between intracranial pressure pulse amplitude and intracranial pressure; AMP, intracranial pressure pulse amplitude; HRsd, standard deviation of heart rate; HRrmssd, root mean square of successive differences of heart rate; LHF, low frequency-high frequency ratio*.

## Discussion

We observed that HT efficacy against ICH is associated with reductions in ICP and ABP. We observed that pre-treatment evidence of efficient CVPR arising from two indices, wPRx and RAC, is associated with effective HT against ICH. In patients without ICH, HT led to increases in ICP and ABP, decreased PVC, and increased HRV.

HT is used against ICH for children with severe TBI. HT is thought to reduce ICH through two mechanisms (23). The first is the establishment of an osmotic gradient sufficient to draw cerebral edema from intraparenchymal tissue into cerebral circulation. The second involves plasma expansion resulting in decreased blood hematoma and reduced blood viscosity. If CVPR mechanisms are intact, this second response would lead to cerebral arteriolar vasoconstriction and a resultant decrease in CABV and ICP. If CVPR is impaired and an insufficient osmotic gradient is developed, HT carries risk of increasing CABV and worsening ICH and cerebral edema. In patients without evidence of ICH, it remains unclear how plasma expansion and the creation of osmotic gradients impacts CVPR, PVC and cerebral oxygen delivery.

Our findings that efficacy of HT against ICH is associated with reduced ICP and ABP is consistent with putative mechanisms. Development of an osmotic gradient reduces intraparenchymal cerebral edema and ICP. Reduced ABP may reflect an appropriate CVPR response to cerebral circulation volume expansion resulting from HT. This is supported by our observation that HT efficacy is associated with lower pre-treatment wPRx and RAC values, reflective of efficient CVPR. We note that PRx values decrease after efficacious treatments and increase after ineffective treatments. This may reflect improved CVPR after efficacious HT, though no changes in other CVPR indices demonstrated significance. Increases in PRx after ineffective treatment likely reflects worsening CVPR. Prospective work investigating the effect of 23.5% hypertonic saline in poor grade subarachnoid hemorrhage patients suggests that it increases cerebral blood flow (24), and work examining mannitol suggests it does not acutely decrease CABV (25). PVC likely differs in young patients with open anterior fontanelles or smaller skulls as compared to older children who may reflect more similar dynamics to adults. Such changes in PVC may reflect changes in treatment efficacy across pediatric ages as we observed.

Our analysis of bolus HT in patients without ICH was performed to gain further insight regarding both the putative mechanisms and implications of HT. Use of bolus HT without ICH is a clinical decision which at our institution may occur to achieve goal serum sodium or osmolality levels desired by the treating team. HT without preceding ICH was associated with increases in ICP and ABP, with each moving oppositely from when HT improves ICH. In treatments without preceding ICH, there was also evidence of decreased HR, increased HRV (HRsd, HRrmssd and LHF Ratio) and decreasing PVC (increasing values of ABP and RAP). In these patients, there is likely insufficient cerebral edema for an osmotic gradient to shift sufficient cerebral edema toward cerebral circulation to reduce ICP. Rather, there is likely plasma expansion resulting in increased CABV, increased ICP and decreased PVC. An increase in ABP along with ICP may reflect impaired CVPR, but this is not clear. Whereas, PRx values increased in these situations, other indices of CVPR decreased. It should be noted that none of these indices of CVPR represent a gold standard for assessment of CVPR. Concern has been raised in prior work as to whether ineffective HT may impair CVPR (3). PRx, regardless of its accuracy in describing CVPR, has been validated as a marker of injury severity in children with TBI (6, 7), and our findings raise question as to whether inefficient use of HT carries risk of disrupting healthy cerebral dynamics and worsening outcomes.

Model-based indices of CVPR, PVC, and HRV derived from MMM data offer clinicians an opportunity to better understand real-time cerebral physiology. What has remained elusive in the implementation of MMM for clinical decision support is whether specific indices of CVPR, PVC, or HRV confer potential benefit for specific treatment strategies in clinical care. In this study, we investigate pre-treatment indices of CVPR, PVC, and HRV as factors that may confer benefit for HT. We observed that lower pre-treatment values for two CVPR indices, wPRx and RAC, that were indicative of efficient CVPR were associated with HT efficacy. PRx represents the most studied index of CA yet its calculation carries significant assumptions. PRx assumes cerebral blood volume as a surrogate of cerebral blood flow, relies on adequate transmission of cerebral blood volume into ICP, assumes that variability of ICP is purely due to extracranial sources, and assumes no incoherence between ABP and ICP. Inadequate transmission of cerebral blood volume into ICP may be common in infants with open anterior fontanelles or children after decompressive craniectomy. In patients with ICH related to malignant cerebral edema and decreasing CPP, ICP variability is related to intracranial sources. The wPRx index investigates wavelet transform phase shifts from ABP and ICP, deriving values when there is strong coherence. The RAC index relates ICP pulse amplitude with CPP, accounting for situations in which there is inadequate transmission of cerebral blood volume into ICP as well as situations in which ICP values are reflective of evolving cerebral edema. Our finding that lower values of wPRx and RAC are associated with treatment responsiveness for ICH suggests that efficient CVPR may confer benefit for HT, and that these indices may offer utility in these circumstances by overcoming unaddressed assumptions made by the PRx calculation.

HT for severe ICH represents the only level II recommendation to offer benefit for pediatric TBI patients. Existing studies that have helped formulate level II recommendations focus on reduction of ICH (26–29) and there are no studies to date demonstrating that HT, or any other therapy, improves functional outcomes. The reasons for the inability to demonstrate such benefit may lie in selection of patients for whom therapy is considered. Existing clinical guidelines support use of HT as a tier II strategy (2, 30) in clinical care without a mechanistic understanding as to whether its use addresses etiologies of ICH at the individualized patient level. Its use focuses on the pathophysiology of cerebral edema, not accounting for circumstances in which ICH may more causally relate to increased CABV, obstructive hydrocephalus, increased metabolic demand, or poor venous drainage. The key component to demonstrating benefit in relation to functional outcomes may lie in the ability to use this therapeutic strategy in select patients demonstrating physiologic characteristics conferring potential to benefit. Our findings suggest that indices demonstrating efficient CVPR may identify those patients for whom HT will reduce ICH. Our findings also suggest that therapeutic benefit of HT in such patients may be associated with improved CVPR and functional outcomes.

This study is limited by a small sample size and its retrospective nature. HT occurred at varied time points in the course of TBI management, and we implemented a working cutoff of ≥50% of bolus HT efficacy to represent subject-level treatment responsiveness. For these reasons, we acknowledge limitations in extrapolating that treatment responsiveness is related to improved outcomes. The analysis methods employed in this study are exploratory techniques that serve for preliminary hypothesis generation. To account for small sample size, we aggregated data regarding both 3% hypertonic saline and mannitol and were unable to assess for differences in efficacy of either therapy. Our findings are limited given high variability of dosing employed for both agents. Because this is a single-center study, findings may not be generalizable across multiple centers. There is a need to conduct larger multi-center studies with standardized dosing and rigorous statistical methodologies to investigate whether model-based indices of CVPR, PVC and HRV may guide therapeutic targeting of HT to reduce ICH and improve functional outcomes.

## Conclusion

After pediatric TBI, efficacy of bolus HT against ICH is associated with pre-treatment physiologic characteristics suggestive of efficient CVPR, and therapy leads to reduction of ICP and ABP. Bolus therapy of HT without preceding ICH may alter cerebral dynamics by increasing ICP and decreasing PVC.

## Data Availability Statement

The raw data supporting the conclusions of this article will be made available by the authors, without undue reservation.

## Ethics Statement

The studies involving human participants were reviewed and approved by Phoenix Children's Hospital Institutional Review Board (IRB #17-469). Written informed consent from the participants' legal guardian/next of kin was not required to participate in this study in accordance with the national legislation and the institutional requirements.

## Author Contributions

JW, MK and BA contributed toward data analysis, preparation, development, and critical review of the manuscript. PA contributed toward preparation, development, and critical review of the manuscript. All authors contributed to the article and approved the submitted version.

## Conflict of Interest

The authors declare that the research was conducted in the absence of any commercial or financial relationships that could be construed as a potential conflict of interest.
