# Supplementary material for: Physiologic Characteristics of Hyperosmolar Therapy After Pediatric Traumatic Brain Injury
Source: Front Neurol. 2021 Apr 20;12:662089. doi: 10.3389/fneur.2021.662089 (PMC8093760; doi:10.3389/fneur.2021.662089)
Supplement: Supplementary file 1 [file Table_1.DOCX]

**Supplemental 1: Model Based Indices of Cerebral Dynamics**

| Cerebrovascular Pressure Reactivity Indices [Higher = Impaired] | | | |  |
| --- | --- | --- | --- | --- |
| Metric | Definition | Signals considered | Calculation | Interpretation |
| PRx | pressure reactivity index | ABP, ICP | Correlation between 30 consecutive 10-sec means of ABP and ICP | Higher PRx = impaired cerebrovascular pressure reactivity |
| PAx | pulse-amplitude index | ABP, ICP | Correlation between 30 consecutive 10-sec means of ABP and ICP pulse amplitude | Higher PAx = impaired cerebrovascular pressure reactivity |
| RAC | correlation coefficient between ICP pulse amplitude and CPP | CPP, ICP | Correlation between 30 consecutive 10-sec means of CPP and ICP pulse amplitude | Higher RAC = impaired cerebrovascular pressure reactivity |
| wPRx | wavelet pressure-reactivity | ABP, ICP | Cosine of wavelet transform phase shift between ABP and ICP | Higher wPRx = impaired cerebrovascular pressure reactivity |
| Heart Rate Variability Indices [Lower = Impaired] | | | |  |
| BRs | Baroreflex sensitivity | ECG, ABP | Modified cross correlation method of R-R intervals and systolic blood pressure | Lower BRS = impaired autonomic function |
| HRsd | standard deviation of HR | ECG | Standard deviation of R-R intervals | Lower HRsd = impaired autonomic function |
| HRrmssd | root mean square of successive differences | ECG | Root mean square of the standard deviation of R-R intervals | Lower HRrmssd = impaired autonomic function |
| LHF | low-high frequency ratio | ECG | Spectral power in R-R low frequency (0.04-0.15 Hz) divided by high frequency (0.15-0.4 Hz) | Lower LHF = impaired autonomic function |
| Pressure Value Compensation Indices | | | | |
| AMP | Amplitude of the ICP waveform | ICP | Lowest point of the ICP waveform subtracted from highest point of ICP waveform | Higher AMP confers decreasing brain compliance |
| RAP | Correlation of AMP and ICP | ICP | Correlation between consecutive 10-sec means of AMP and ICP | RAP=0: good compensatory reserve;  RAP=1: poor compensatory reserve  RAP<0: Deranged cerebrovascular reactivity |

Legend: PRx, pressure reactivity index; PAx, pulse amplitude index; RAC, correlation coefficient between intracranial pressure pulse amplitude and cerebral perfusion pressure; wPRx, wavelet-pressure reactivity; HRsd, standard deviation of heart rate; HRrmssd, root mean square of successive differences of heart rate; LHF, low frequency-high frequency ratio; ABP, arterial blood pressure; ICP, intracranial pressure; CPP, cerebral perfusion pressure; ECG, electrocardiogram; AMP, intracranial pressure pulse amplitude; RAP, correlation coefficient between intracranial pressure pulse amplitude and intracranial pressure sec, seconds; Hz, hertz.
